# HLM-MOCAP: A motion capture dataset of context-dependent human arm motion for human-like robot motion generation

**DOI:** 10.1016/j.dib.2026.112849

**Published:** 2026-05-12

**Authors:** Bsher Karbouj, M. Subie Hiswani, Ediz Demir, Jörg Krüger

**Affiliations:** Technical University of Berlin, Department of Industrial Automation Technology, Berlin Germany

**Keywords:** Human-like motion, Motion capture, Human arm motion, Human–robot collaborationL, Learning from demonstration

## Abstract

HLM-MOCAP is a motion capture dataset of context-dependent human arm movements designed to support the generation of human-like robot trajectories in industrial and collaborative settings. Data were recorded in a laboratory environment using an eight-camera infrared motion capture system (Vicon MX T10) operating at 200 Hz with 1120 × 896 px resolution and passive 1.5 mm retroreflective markers. Forty healthy adults (29 male, 11 female; broad age range from late adolescence to adulthood) performed a set of seven upper-limb tasks at a standardized workstation with marked start and target locations and physical objects of varying weight and size. The tasks comprise point-to-point transport to different distances and directions, sequential reaching to multiple targets, two trajectory-tracing tasks (zigzag and circle) with the index finger, a grasp-and-place task with arm crossing, a reaching task with load, and a high-precision placement task using small screws. Each participant completed 54 movement executions (with task-specific repetition counts), yielding 2160 recorded movement trials in total. The dataset includes raw 3D marker trajectories for all trials, segmented to active movement phases based on an objective velocity threshold, and processed versions after gap filling of short occlusions, time normalization to 0–100 % movement progress, and Savitzky–Golay filtering for derivation of stable velocity and acceleration profiles. Data are organized as comma-separated files with participant identifiers, task labels, target indices, object properties, and repetition indices, accompanied by scripts for loading and basic preprocessing. The resource can be reused for developing and benchmarking methods for human-like robot motion planning, learning from demonstration, and trajectory generation, as well as for analyzing how distance, sequence structure, load, and precision demands shape human arm kinematics in the context of human–robot collaboration.

Specifications Table

**Table:** 

Subject	Computer Sciences
Specific subject area	*Robotics; Human–Robot Collaboration; Motion Capture and Human Motion Analysis; Learning from Demonstration*
Type of data	CSV files (raw and processed motion capture trajectories; interpolated, trimmed, and time-normalized trajectories; per-subject and across-subject averages; cluster-wise averages; kinematic summaries; deviation/percentage tables) Graphs / images (PNG plots of trajectories, velocity and acceleration profiles, overlay plots, heatmaps, boxplots, and bar charts) Code (Python scripts for data formatting, cleaning, interpolation, averaging, clustering, and visualization)
Data collection	Human arm motions were captured in a laboratory with an infrared motion capture system (Vicon MX, 8 cameras, 200 Hz) using retro-reflective markers on the dominant arm while volunteers performed standardized reaching and manipulation tasks at a fixed workstation. Raw 3D marker trajectories were exported from Vicon Nexus and processed in Python (gap filling of short occlusions, velocity-based movement segmentation, time normalization to 0–100 %).
Data source location	*Technical University of Berlin*
Data accessibility	Repository name: Mendeley Data Data identification number: 10.17632/6nwppg5syn.1 Direct URL to data: https://data.mendeley.com/datasets/6nwppg5syn/1
Related research article	None

## 1 Value of the Data

• The dataset provides synchronized motion capture recordings of human arm trajectories across multiple reaching, manipulation, and precision tasks with varied distances, paths, loads, and constraints, enabling systematic analysis of how task context shapes upper-limb kinematics.
• The data can be reused to train, validate, and benchmark algorithms for human-like robot motion generation, including learning from demonstration, trajectory optimization, and motion prediction in industrial and collaborative robotics.
• The dataset includes raw motion capture exports in CSV format and processed CSV trajectories (gap-filled, segmented, and time-normalized), together with example Python code, allowing researchers to either reproduce the full processing pipeline or directly build on ready-to-use kinematic features.
• The structured metadata (participant IDs, task labels, target indices, object properties, repetition numbers) facilitate controlled comparative analyses, cross-subject averaging, clustering of movement patterns, and integration with additional sensing modalities or simulated robot experiments.

## 2 Background

In collaborative and flexible manufacturing, there is increasing interest in generating robot motions that resemble human arm movements to improve predictability, comfort, and acceptance of collaborative robots. Recent work on human-like arm motion generation and learning-based control emphasizes the need for high-quality human motion data to support such approaches [1,2]. At the same time, learning from demonstration is promoted as a practical way to program robotic manipulators for industrial tasks, reducing manual trajectory tuning and simplifying deployment [3]. Several open datasets provide human motion data for robotics, including upper-limb or whole-body recordings and reaching movements with additional sensing such as gaze [[4], [5], [6]]. However, these resources mainly target activities of daily living, virtual reality environments, or specific robot platforms, and offer limited coverage of constrained single-arm table-top tasks that resemble collaborative assembly with cobots. Against this background, the dataset described in this article compiles motion capture recordings of human arm trajectories in a set of standardized reaching, manipulation, and precision tasks designed to support data-driven generation of human-like robot trajectories for collaborative assembly scenarios.

## 3 Data Description

The dataset is organized into a hierarchical folder structure that documents all stages of data collection, preprocessing, and derived outputs. All raw, intermediate, and processed files are included, and each processing step produces its own dedicated subdirectory. Table 1 provides an overview of the complete folder structure, including the main content of each folder, the associated processing stage, and the units used for the trajectories and derived features. This structure enables full traceability of data transformations and allows the workflow to be reproduced without ambiguity. All motion capture files follow a consistent format after preprocessing. Each CSV file contains a sequential frame index and three-dimensional coordinates for each recorded marker. Columns adhere to the naming scheme <markerID>_X, <markerID>_Y, and <markerID>_Z. Marker identifiers range from 1 to 5, with specific experiments using subsets of these markers. File names encode both the participant identifier and the experimental condition using predefined keywords (e.g., circle, ptp, zigzag, grasp, weight, precision, sequential), allowing automated assignment of trials to task categories. The directory layout clearly separates raw input data from all subsequent processing stages. Each transformation step generates its own output folder, ensuring that all intermediate states of the data are preserved and accessible for inspection or re-analysis.

**Table 1:** Overview of the HLM-MOCAP dataset structure.

Category	Folder/File	Key contents	Description	Unit	Stage
Raw and Preprocessed Data	Daten_Raw/	Original files	Unformatted CSV exports from motion‑capture system.	–	Raw input
	Daten_Raw_Formatted/	Standardized raw files	Unified delimiters, column structures, consistent metadata.	–	Formatting
	Daten_Raw_Clean/	Corrected trajectories	Marker correction, artifact removal, coordinate normalization.	mm, frames	Cleaning
	Daten_Raw_Interpolated/	Gap-filled trajectories	Missing frames reconstructed via interpolation.	mm, frames	Interpolation
	Daten_Trimmed/	Trimmed trajectories	Passive or irrelevant portions removed.	mm, frames	Trimming
Processed and Aggregated Data	Daten_Averaged_1M/	Participant averages	Averaged trajectories per participant and condition.	mm, samples	Aggregation
	Final_Averages_1M/	Experiment averages	Group-level mean trajectories.	mm, samples	Final aggregation
	Final_Cleaned/	Postprocessed data	Smoothed and fully processed final trajectories.	mm, samples	Postprocessing
Visualization Outputs	Plots/	Generated figures	Movement profiles, diagnostics, workflow figures.	PNG, PDF	Visualization
	Single_Plots/	Single-trial figures	Plots of manually selected single-trial data.	PNG, PDF	Visualization
Specialized and Segmented Data	Clustered/	Grouped datasets	Data split into demographic or experimental segmentation.	–	Segmentation
	Single_Selected/	Selected trials	Manually chosen individual trajectories.	mm, frames	Selection
Reference and Utility Files	Deviation references /	Reference paths	Template movements (e.g., circular, zigzag) for alignment.	mm	Reference
	visualize_marker_ trajectories.py	Visualization script	Generates 3D marker trajectory visualizations.	–	Utility
	Step1–Step11	Processing scripts	Full preprocessing and analysis pipeline.	–	Pipeline

## 4 Experimental Design, Materials and Methods

### 4.1 Participants and experimental design

Forty healthy adult volunteers (29 male, 11 female) without known motor impairments or relevant neurological or musculoskeletal disorders participated in the motion capture study. Age was broadly distributed, with most participants between 18 and 35 years. All participants were informed about the purpose and procedure of the recording session and provided written informed consent prior to data collection. The experiment followed a within-subject design: every participant completed the full set of seven arm-movement tasks. The order of tasks was fixed and identical for all participants. For each task, a predefined number of repetitions was recorded: five repetitions per task in most conditions, with 15 repetitions for the point-to-point transport task, 10 repetitions for the grasp-and-place task (five from each initial hand position), and nine repetitions for the precision placement task. This resulted in 54 recorded movements per participant and a total of 2160 movement executions in the dataset.

### 4.2 Experimental setup and materials

Recordings were conducted in a motion capture laboratory using eight infrared cameras (Vicon MX T10) mounted in the corners and midpoints of the long walls around the measurement volume. At the beginning of each session, a static T-pose calibration was performed, and additional markers on the corners of the experimental table defined the table plane within the laboratory coordinate system. Experiments took place at a standardized table-top workstation. Start and target positions were physically marked on the table surface, and printed templates for trajectory-tracing tasks were placed directly on the tabletop. Participants interacted with a set of physical objects whose dimensions and masses are documented in the dataset: a plastic cube (55 × 55 × 55 mm, ≈120 g), a hollow plastic cylinder (diameter 70 mm, height 115 mm, ≈160 g), a weighted cylinder of approximately 2 kg, and three M4 screws of different lengths (20, 25, and 30 mm).

### 4.3 Motion capture instrumentation and marker placement

**Fig. 1:**
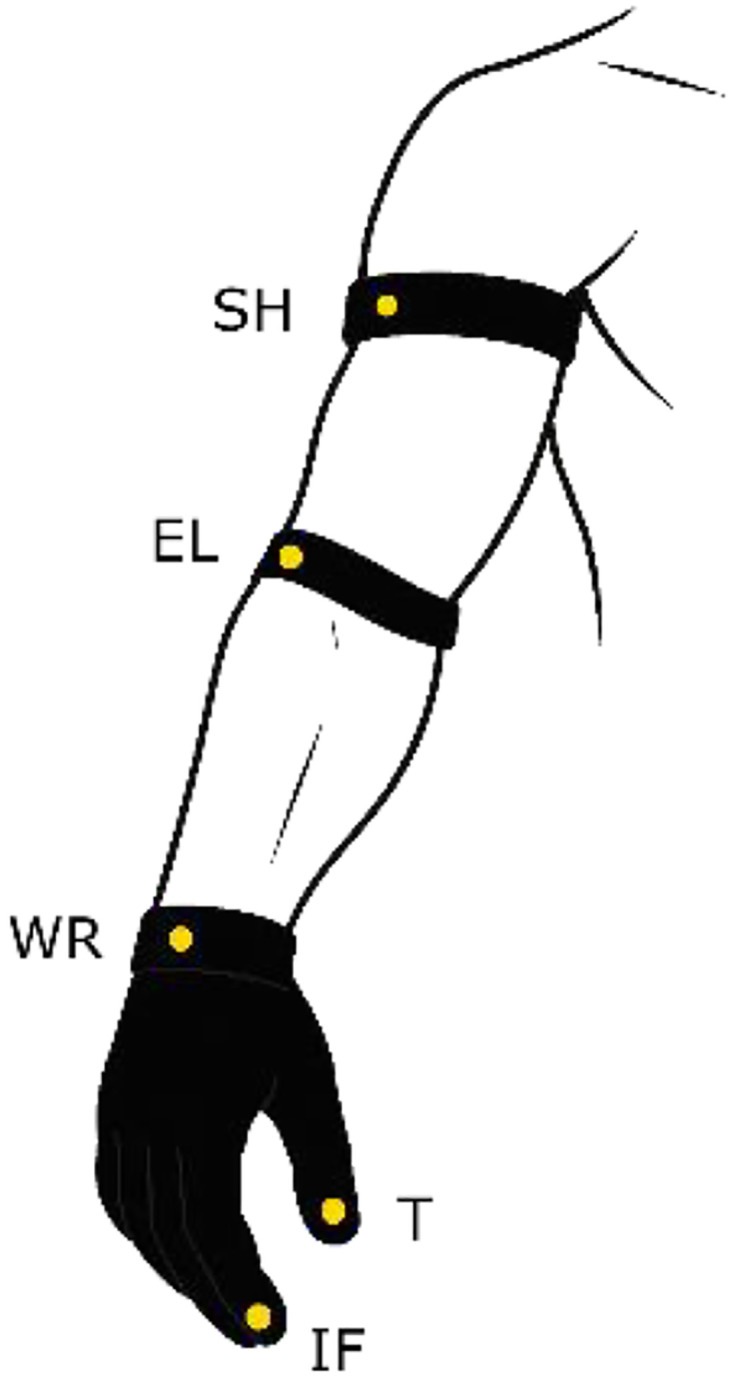
Motion capture setup for the HLM-MOCAP dataset.

Markers were attached to tight-fitting gloves and Velcro straps to ensure stable tracking (see Fig. 1). Depending on the task, up to five anatomical landmarks on the dominant arm were tracked: thumb tip, index fingertip, dorsal wrist, lateral elbow, and acromion region at the shoulder. Marker configurations were adapted between tasks according to the primary kinematic focus, for example using the index fingertip for trajectory-tracing tasks and the full five-marker configuration for grasping, weighted transport, and precision placement tasks. After completion of all tasks, all markers were removed. The marker configuration was adjusted to the requirements of each task, and the resulting combinations of thumb, index finger, wrist, elbow, and shoulder markers are summarized in Table 2.

**Table 2:** Marker placement for the HLM-MOCAP tasks.

Task	T	IF	WR	EL	SH
T1 - PTP					
T2 – Sequential					
T3 – Zigzag					
T4 – Circle					
T5 – Grasp / Place					
T6 – Weight					
T7 – Precision					

*T* = Thumb, IF = Index Finger, WR = Wrist, EL = Elbow, SH = Shoulder.

### 4.4 Task protocol

Upon arrival in the lab, participants received a brief verbal explanation of the goal and structure of the session, followed by an introduction to the motion capture system and the experimental setup. Demonstrations of the tasks were generally avoided to prevent imitation effects; only in cases of misunderstanding was a short demonstration provided. Written consent was obtained before any recording started. A complete recording session per participant took approximately 15 min. Each participant then performed the seven tasks in a fixed order:

• T1 – Point-to-point transport: transport of a plastic cube from a fixed start position to one of three targets at different distances and lateral positions (center, front-right, front-left). Targets were executed in blocks of five repetitions per target.
• T2 – Sequential transport: sequential movement of the same cube through five targets in a predefined order (1–2–3–2–1), using the same coordinates as in T1.
• T3 – Zigzag trajectory tracing: the index finger followed a printed zigzag line on the tabletop from a lower start point to an upper end point.
• T4 – Circular trajectory tracing: the index finger traced a closed circular contour on the tabletop along a printed template; the direction of traversal was not constrained.
• T5 – Grasp and place: participants grasped a hollow cylinder from the table and placed it at a designated target. This was performed twice: once with the hand starting to the right of the cylinder and once with the hand starting to the left, inducing arm crossing in the latter condition.
• T6 – Weighted transport: participants grasped a weighted cylinder (≈2 kg) and transported it from a start position to a single target.
• T7 – Precision placement: participants inserted three screws of different lengths multiple times into matching small holes, emphasizing fine motor control and endpoint accuracy.

## Limitations

The dataset was recorded in a single motion capture laboratory using one Vicon system and a standardized table-top workstation, so the data primarily reflect this specific environment and setup. Participants are healthy adults, mostly young, and only the dominant arm was instrumented with a limited set of markers; full-body motion, non-dominant arm movements, and detailed hand joint kinematics are not included.

The task set focuses on constrained, single-arm reaching, manipulation, and precision placement at a fixed workstation and does not cover bimanual activities, whole-body locomotion, or direct physical interaction with collaborative robots. Motion capture data can contain short marker occlusions; these were handled by interpolation within the provided preprocessing pipeline, while trials with more severe occlusions were excluded from the processed releases. Finally, the processed trajectories are based on specific preprocessing choices (gap filling, trimming, time normalization, and smoothing). Users who require alternative representations or parameter settings may wish to reprocess the raw exports with their own pipelines.

## Ethics Statement

All participants were adults and provided written informed consent prior to participation, including consent for the collection and anonymized sharing of motion capture and behavioral data. According to the institutional guidelines of the Technical University of Berlin, such non-interventional behavioral studies do not require formal review by an ethics committee.

## Credit Author Statement

**Bsher Karbouj:** Conceptualization, Supervision, Methodology, Writing - original draft, Writing - review & editing. **M Subie Hiswani:** Conceptualization, Data curation, Methodology, Software, Writing - original draft, Writing - review & editing. **Ediz Demir:** Investigation, Data curation. **Jörg Krüger:** Supervision, Writing - review & editing.

## Data Availability

Mendeley DataHLM-MOCAP: A Motion Capture Dataset of Context-Dependent Human Arm Motion for Human-Like Robot Motion Generation (Original data)
